# Extended Postinterventional Tumor Necrosis—Implication for Outcome in Liver Transplant Patients with Advanced HCC

**DOI:** 10.1371/journal.pone.0053960

**Published:** 2013-01-22

**Authors:** Arno Kornberg, Ulrike Witt, Edouard Matevossian, Bernadett Küpper, Volker Aßfalg, Alexander Drzezga, Norbert Hüser, Moritz Wildgruber, Helmut Friess

**Affiliations:** 1 Department of Surgery, Klinikum rechts der Isar, Technical University, Munich, Germany; 2 Department of Surgery, Klinikum Bad Berka, Bad Berka, Germany; 3 Department of Nuclear Medicine, Klinikum rechts der Isar, Technical University, Munich, Germany; 4 Institute of Interventional Radiology, Klinikum rechts der Isar, Technical University, Munich, Germany; The University of Hong Kong, Hong Kong

## Abstract

**Background:**

Locoregional interventional bridging therapy (IBT) is an accepted neoadjuvant approach in liver transplant candidates with hepatocellular carcinoma (HCC). However, the prognostic value of IBT in patients with advanced HCC is still undefined.

**Aim:**

The aim of this trial was to evaluate the impact of postinterventional tumor necrosis on recurrence-free long-term survival after liver transplantation (LT) in patients with HCC, especially focusing on those exceeding the Milan criteria on pretransplant radiographic imaging.

**Patients and Methods:**

A total of 93 consecutive liver transplant candidates with HCC were included in this trial. In 36 patients, tumors were clinically staged beyond Milan criteria prior LT. Fifty-nine patients underwent IBT by transarterial chemoembolization or radiofrequency ablation pretransplantation. Postinterventional tumor necrosis rate as assessed at liver explant pathology was correlated with outcome post-LT.

**Results:**

There was no significant difference in 5-year tumor-free survival rate between the IBT- and the non-IBT subpopulation (78% versus 68%, *P* = 0.25). However, tumor response following IBT (≥50% tumor necrosis rate at explant pathology) resulted in a significantly better outcome 5 years post-LT (96%) than tumor non-response to IBT (<50% tumor necrosis rate at explant pathology; 21%; *P*<0.001). Five-year recurrence-free survival rate was 80% in Milan Out patients with extended post-IBT tumor necrosis versus 0% in Milan Out patients without tumor response to IBT (*P*<0.001). None of macromorphological HCC features, but only the absence of increased ^18^F-fluoro-deoxy-glucose (^18^FDG) uptake on pretransplant positron emission tomography (PET) was identified as independent predictor of postinterventional tumor response (*P*<0.001).

**Conclusion:**

Our results implicate that extended postinterventional tumor necrosis promotes recurrence-free long-term survival in patients with HCC beyond standard criteria. Pretransplant PET assessment may identify those patients with advanced HCC that will benefit from post-IBT tumor response and may, thereby, achieve excellent posttransplant outcome.

## Introduction

Hepatocellular carcinoma (HCC) is the most common primary tumor of the liver and its incidence is expected to rise continuously. Cirrhosis is present in approximately 90% of the cases and frequently limits curative liver resection. Liver transplantation (LT) has the advantage to remove the tumor and the underlying cirrhosis. Furthermore, it is able to restore normal hepatic function [1]. However, early results were discouraging with high tumor recurrence rates and dismal patient survival because of advanced tumor stage [1], [2]. The implementation of the Milan criteria (one tumor nodule up to 5 cm, maximum of 3 tumor nodules each up to 3 cm, no macroscopic vascular invasion or extrahepatic disease) in 1996 by Mazzafero et al. has established LT as standard therapy in patients with early stage HCC in liver cirrhosis [3]. Patients with tumors selected according these standard criteria may achieve a 5-year recurrence-free survival rate about 70%, which is an extraordinary outcome data in oncological surgery [4], [5]. They have been adopted by the United Network for Organ Sharing and by the Eurotransplant Foundation as standard criteria for listing patients with HCC. In both transplant organizations, liver allocation is currently based on the Model for End Stage Liver Disease [MELD), assigning exceptional priority points for patients with tumors that are meeting the Milan criteria, so that timely LT can be performed [6]. In recent years, however, several groups have argued that the Milan criteria might be too restrictive, and exclude a high number of patients from potentially curative LT [4], [7], [8], [9], [10]. In 2001, colleagues from the University of California San Francisco have defined the UCSF criteria (one single tumor up to 6.5 cm, or up to 3 tumors with the largest being 4.5 cm in diameter, with a total tumor diameter <8 cm) as reasonable new macromorphological tumor burden, implementing locoregional interventional bridging treatments (IBT) prior LT [8]. Subsequently, the UCSF criteria have been adopted in several transplant centers as standard for indicating LT.

In recent years, IBT by transarterial chemoembolization (TACE), radiofrequency ablation (RFA) or percutaneous ethanol injection has gained wide acceptance around the world [9]–[12]. Apart from tumor downstaging into accepted criteria [13], [14], [15], bridging to LT in order to reduce the risk of tumor-related patient drop-out and posttransplant tumor recurrence is another clinical approach [12], [13], [16], [17]. Significant reduction of vital tumor load should, therefore, be the crucial outcome variable of this procedures [17]. Nevertheless, data about the prognostic value of postinterventional tumor necrosis in the transplantation setting are still very limited.

In view of these considerations, the purpose of our study was to analyze the impact of IBT-induced tumor necrosis on posttransplant long-term outcome in liver transplant patients with HCC, especially focusing on those tumors exceeding the Milan criteria on pretransplant clinical staging.

## Patients and Methods

### Patient characteristics

Between 1996 and 2008, 114 patients with HCC in liver cirrhosis were listed for LT (Table 1). Nine patients presenting incidental small HCC assessed only at pathological specimen were not included in the analysis.

**Table 1:** Characteristics of all HCC patients listed for HCC (n = 114).

*Variable*	*Value*
**Recipient Age (y)**	
Mean ± STD	58.9±6.9
Range	38–71
**Sex (n)**	
Male	69
Female	45
**Aetiology of liver disease (n)**	
Alcoholic	62
Viral	33
Cholestatic	3
Metabolic	2
Autoimmune	8
Hemochromatosis	6
**Child status**	
A	48
B	40
C	26
**IBT pre-LT (n)**	
TACE	63
RFA	8

IBT: locoregional interventional bridging therapy.

TACE: transarterial chemoembolization.

RFA: radiofrequency ablation.

**Figure 1:**
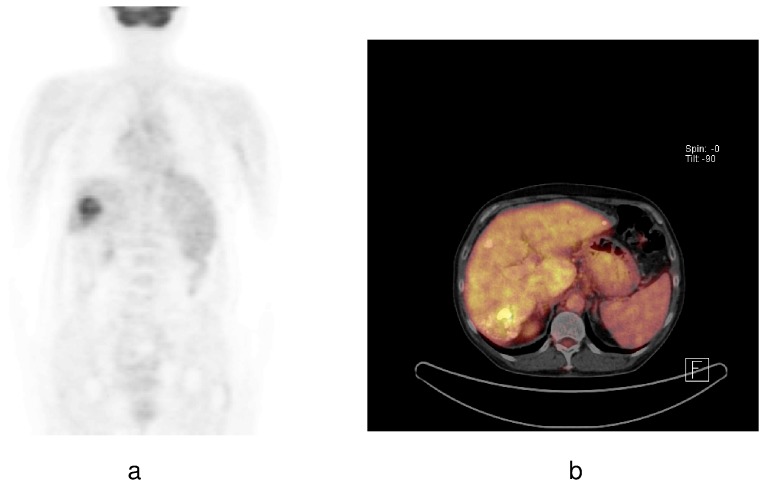
HCC with increased ^18^F-FDG uptake on ***a*** pretransplant PET scan and ***b*** PET-CT scan were classified as PET +.

Diagnosis of HCC was established by clinical staging, including ultrasonography, dynamic computed tomography (CT), contrast-enhanced magnetic resonance tomography (MRI), and alpha-fetoprotein (AFP) measurements. Furthermore, whole-body ^18^F-fluoro-deoxy-glucose (^18^FDG) positron emission tomography (PET) was performed for clinical staging minimum once pre-LT and before initiating IBT in all patients. According to the glucose metabolism on ^18^F-FDG PET, tumors were classified as PET − (no increased FDG uptake as compared to the surrounding liver tissue) or PET + (increased FDG uptake as compared to the surrounding liver tissue; Fig. 1), as previously described [18], [19]. We did not routinely perform preoperative tumor biopsy.

### Patient listing and drop out criteria

Patient listing was based on the Milan criteria since 1996 [3]. After the introduction of the MELD system in the Eurotransplant region in December 2006, patients with HCC fulfilling the Milan criteria on clinical staging have received exceptional MELD priority points [6]. Macromorphological tumor progression beyond the Milan or UCSF burden did not automatically result in patient drop out from the waiting list at our center. However, those patients have lost their exceptional MELD priority points and were considered for LT with marginal allografts or living donor liver transplants. Macroscopic tumor invasion into a major vascular branch, lymph node metastases, extrahepatic tumor spread and severe tumor-related symptoms disqualified for LT [20]. Based on final pretransplant radiographic staging of viable tumor areas, patients were classified according the Milan and UCSF criteria (Milan In versus Milan Out; UCSF In versus UCSF Out) [3], [5], [8], [9].

### Locoregional interventional bridging therapy pre-LT

Neoadjuvant IBT pretransplantation was implemented at our center in 1999. All liver transplant candidates were discussed at a multidisciplinary liver conference, where treatment plans were established. Based on clinical condition, liver function and tumor topography/morphology, TACE was the preferred interventional procedure. It was performed in a standardized manner [21]. Briefly, an aortography was carried out by catheterization of the femoral artery to illustrate the coeliac trunk and the mesenteric arteries. Subsequently, the tumor feeding arteries were selected and catheterized as selectively as possible. A mixture of epirubicin and lipiodol (20 ml) was infused under real time fluoroscopic control. The following day, liver function tests were analyzed and the arterial supply of the liver was controlled by duplexsonography. Follow-up contrast CT scans were performed within 6 weeks post-intervention for tumor re-staging. Depending on liver function and radiographic imaging, a maximum of 6 TACE procedures have been planned.

Radiofrequency ablation (RFA) of the tumor was critically discussed, if patients appeared to be ineligible for TACE, either for liver dysfunction and/or for morphology/topography of the tumor. RFA was performed percutaneously and CT-guided under general anaesthesia [22], [23]. A maximum of 3 RFA procedures were indicated using monopolar perfused electrodes (HITT®, Berchtold Integra, Tübingen, Germany). Clinical response of IBT was monitored by MRI evaluation of the liver within 6 weeks.

### Explant histopathology and postinterventional tumor necrosis

At definitive pathological examinations of the explant liver, HCC was confirmed in all cases. Tumors were examined according to size, number, total tumor diameter, stage, lymphatic and vascular invasion, respectively. Tumor differentiation was determined according to Edmondson and Steiner's grading system. Histopathologic tumor staging was assigned by co-operation of the surgery and pathology staff based on clinical and pathologic data according to the 5^th^ edition of the Tumor, Node, Metastasis/International Union Against Cancer criteria of 1997.

**Figure 2:**
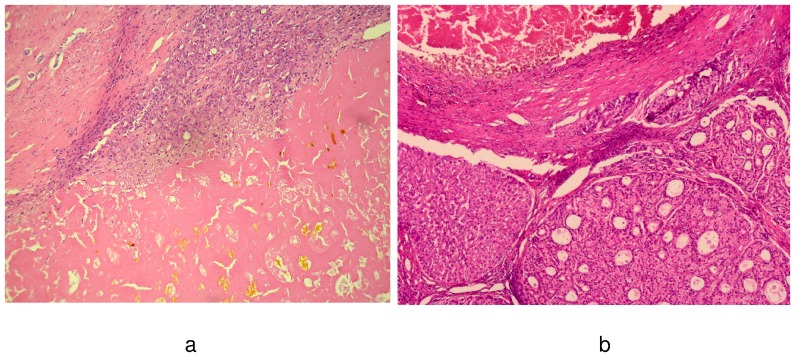
This figure demonstrates the micrographs (HE, 50×; 70×) of a post-IBT tumor responder with near complete postinterventional tumor necrosis (a), and of a tumor non-responder to IBT with necrotic tumor areas next to pseudoglandular HCC nodules (b), respectively.

The postinterventional tumor necrosis rate was defined as the proportion of the necrotic region to the total presumed tumor area. It was categorized as “complete” (no viable tumor), greater than 75%, between 50% and 75%, or less than 50%. “Tumor response” to IBT was postulated if a minimum tumor necrosis rate of 50% was assessed, while tumor necrosis rate <50% indicated “tumor non-response” to IBT (Fig. 2).

### Immunosuppressive therapy and posttransplant tumor surveillance

Immunosuppression consisted of a calcineurin inhibitor based regimen (cyclosporine A [CsA] versus tacrolimus [Tac]), either augmented with azathioprine or mycophenolate mofetil and prednisone. Corticosteroids were completely tapered in all patients within 6 months with exception of those with autoimmune hepatitis. Ultrasonography of the liver allograft and AFP level measurement were performed every three months post-LT. In addition, patients underwent CT/MRI evaluation every 6 months in the first posttransplant year and minimum yearly thereafter, or in the case of an increasing AFP level.

### Assessment of prognostic variables

All data were collected in a prospective database and retrospectively analyzed. Formal approval of the local ethics committee was not required due to the studies' retrospective character and the fact that all applied procedures (TACE, RFA, liver transplantation) have already been established therapeutical interventions (consultation of the local ethical institution). Before a liver transplant candidate has been put on the waiting list, he underwent concise medical information about the upcoming interventional treatments and LT. Furthermore, by a written consent all liver transplant patients have accepted that data of their clinical follow-up will be used for academic studies.

The impact of the following variables on recurrence-free long-term survival was analyzed:

Pretransplant (clinical) variables:

Donor and recipient age, sex, Child status, AFP-level, size and number of tumor nodules (radiographic imaging), Milan and UCSF criteria (radiographic imaging), ^18^F-FDG tumor uptake on PET

Posttransplant variables:

Microvascular and lymphatic invasion, tumor differentiation, tumor response to IBT (explant pathology), immunosuppressive therapy (CsA versus Tac).

### Statistical analysis

The software SPSS 17.0 for Windows was used for statistical analyses. Clinical and histopathologic variables were correlated with frequencies of tumor recurrence using χ^2^ test and logistic regression. Overall and recurrence-free survival rates were determined according to the Kaplan-Meier method. Patient survival in different groups was compared using the log-rank test. The value of clinical variables for predicting postinterventional tumor response was assessed by χ^2^ test and logistic regression.

Variables with a significant prognostic impact on univariate analysis (*P*<0.05) were entered into a stepwise multivariate analysis (Cox multiple stepwise regression model). Only clinical but not histopathologic variables defining the Milan and UCSF criteria were included in the analysis.

## Results

### Patient and tumor characteristics

Twenty-one patients dropped from the waiting list, 14 of them as a result of tumor progression. Finally, 93 patients underwent LT (Table 2).

**Table 2:** Patient and tumor characteristics of all liver recipients (n = 93) according to pretransplant IBT.

*Variable*	*IBT*	*non-IBT*	*P-value*
	(n = 59)	(n = 34)	
Age recipient at LT (y)	58.1±6.7	58.4±7.5	0.84
Age donor at LT (y)	50.8±14.1	46.2±14.5	0.14
Child status (n)			0.86
A	30	18	
B or C	29	16	
Waiting time prior LT (months)	6.1±3,2	6.6±4.0	0.54
Liver allograft (n)			0.37
deceased	52	29	
living related	´7	6	
Mean AFP level at LT (ng/ml)	1012±6127	903±2509	0.92
*Mean number tumor nodules (n, range)	2.2±1.6 (1–8)	1.5±1.1 (1–5)	0.03
*Mean diameter largest tumor nodule (cm)	4.0±2.1	4.6±3.5	0.30
*Mean total tumor diameter (cm)	6.5±4.0	6.2±4.5	0.69
*Milan status (n)			0.71
In	37	20	
Out	22	14	
*UCSF status (n)			0.91
In	41	24	
Out	18	10	
PET + status (n)	40	18	0.51
Poor tumor differentiation (n)	9	8	0.32
Major vascular invasion (n)	1	3	0.11
Lymphatic vascular invasion (n)	8	9	0.12
Immunosuppression (n)			0.02
CsA	18	19	
Tac	41	15	

* based on final pretransplant clinical staging.

AFP: alpha-fetoprotein.

Tac: tacrolimus.

PET: positron emission tomography.

UCSF: University of California San.

CsA: cyclosporine A.

Francisco.

There were 61 male and 32 female liver recipients. Mean patient age at LT was 58.2 years (range: 38–71 years). Eighty patients have received a full size liver allograft, while 13 patients underwent living donor liver transplantation. At final pretransplant radiographic staging, 36 patients demonstrated HCC beyond the Milan criteria and 28 patients revealed tumors exceeding the UCSF criteria, respectively (Table 2).

Fifty-nine patients underwent pretransplant IBT (63.4%). Fifty-one of them were treated by TACE (between 1 and 5 TACE procedures), while 8 patients underwent RFA therapy, respectively. Mean number of tumor nodules on pretransplant clinical staging and number of patients under Tac-based immunosuppression were significantly higher in the IBT-subpopulation (Table 2).

### Tumor response to IBT at explant pathology

Tumor response to IBT (≥50% tumor necrosis rate) was confirmed in 44 patients (complete tumor necrosis n = 1; tumor necrosis rate >75%: n = 18; tumor necrosis rate 50%–75%: n = 25). In contrast, 15 liver recipients were classified as tumor non-responders to IBT (25.4%).

### HCC recurrence and survival rates

Current overall survival is ranging between 5 and 162 months posttransplantation (mean: 64.3±44.2 months).

Five-year overall and recurrence-free survival rates of the entire study population (n = 93) were 76% and 74%, respectively.

**Figure 3:**
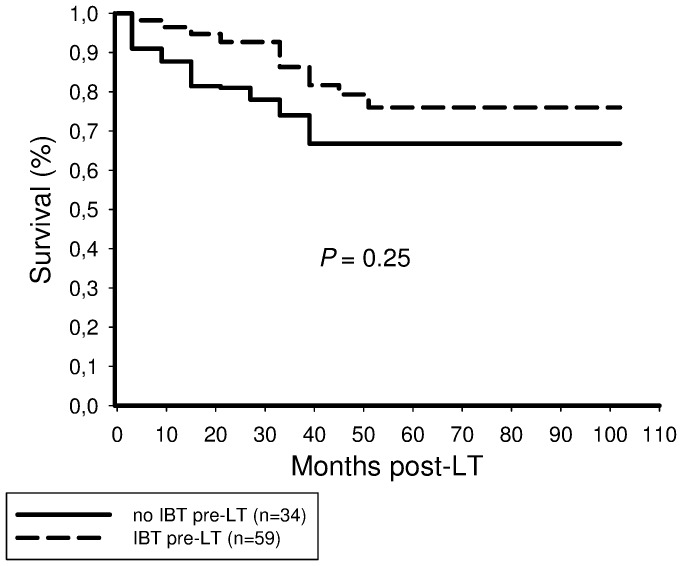
There was no significant difference in recurrence-free survival between patients receiving and those not receiving pretransplant IBT (*P* = 0.25).

There was no significant difference in 5-year tumor-free survival between the IBT (78%) and the non-IBT (68%) recipients (*P* = 0.25; Fig. 3).

Twenty-one patients developed tumor recurrence (22.6%), 11 patients in the IBT-group (18.6%) and 10 patients in the non-IBT-population (29.4%, *P* = 0.23). Tumor recurrence rates tended to be lower by IBT in both, patients with HCC meeting and exceeding the Milan and UCSF criteria, respectively (Table 3).

**Table 3:** Tumor recurrence rates in patients meeting and exceeding the Milan/UCSF criteria, according to IBT.

	*IBT (n = 59)*	*non-IBT (n = 34)*	*P-value*
**Milan In (n; %)**	4/37 (10.8%)	3/20 (15%)	0.65
**Milan Out (n; %)**	7/22 (31,8%)	7/14 (50%)	0.28
**UCSF In (n; %)**	4/41 (9.8%)	4/24 (16.7%)	0.41
**UCSF Out (n; %)**	7/18 (38.9%)	6/10 (60%)	0.28

UCSF: University of California San Francisco.

Finally, 44 patients turned out to be tumor responders to IBT, while postinterventional tumor necrosis rate was below 50% in 15 liver recipients. Only one of 44 responders (2.3%) but 10 of 15 non-responders (66.7%) suffered from posttransplant tumor recurrence (*P*<0.001).

**Figure 4:**
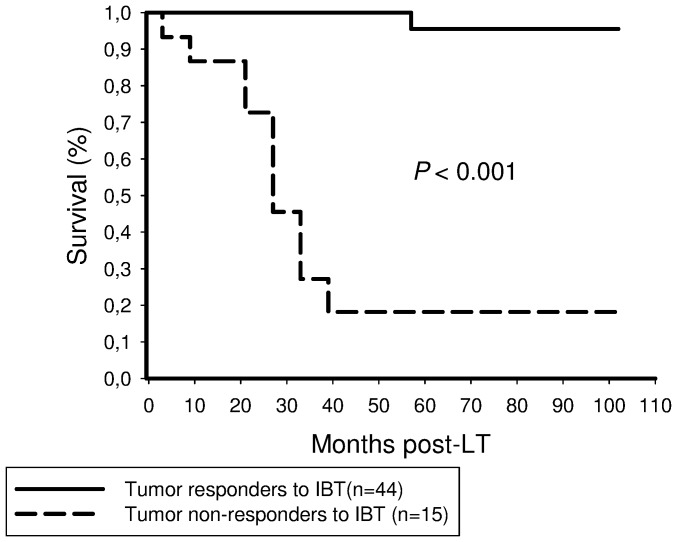
Tumor responders to IBT had a significantly better 5-year recurrence-free survival probability (90%) than patients without tumor response to IBT (21%; *P*<0.001).

Post-IBT tumor responders to IBT had a significantly better recurrence-free survival rate after 5 years than non-responders to IBT (96% versus 21%; Fig. 4).

**Figure 5:**
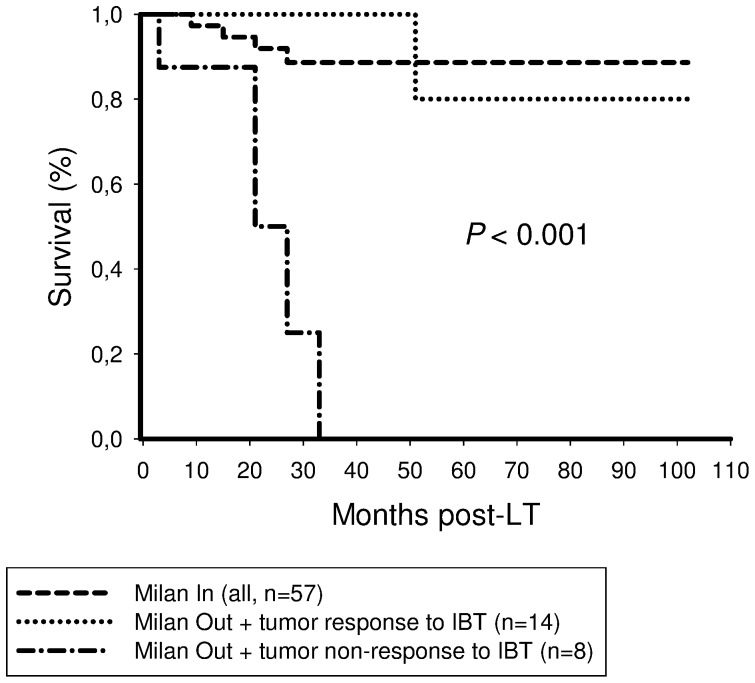
There was no significant difference in 5-year recurrence-free survival rate between Milan In recipients (87%) and Milan Out patients demonstrating post-IBT tumor response (80%). In contrast, none of Milan Out recipients without tumor response to IBT have survived 5 years post-LT (*P*<0.001).

Patients with Milan Out tumors on clinical staging but demonstrating postinterventional tumor response had a 5-year recurrence-free survival rate of 80%. This was comparable to the Milan In group of our trial (86.6%), but significantly better than in Milan Out patients without tumor response to IBT (0%; Fig. 5).

**Figure 6:**
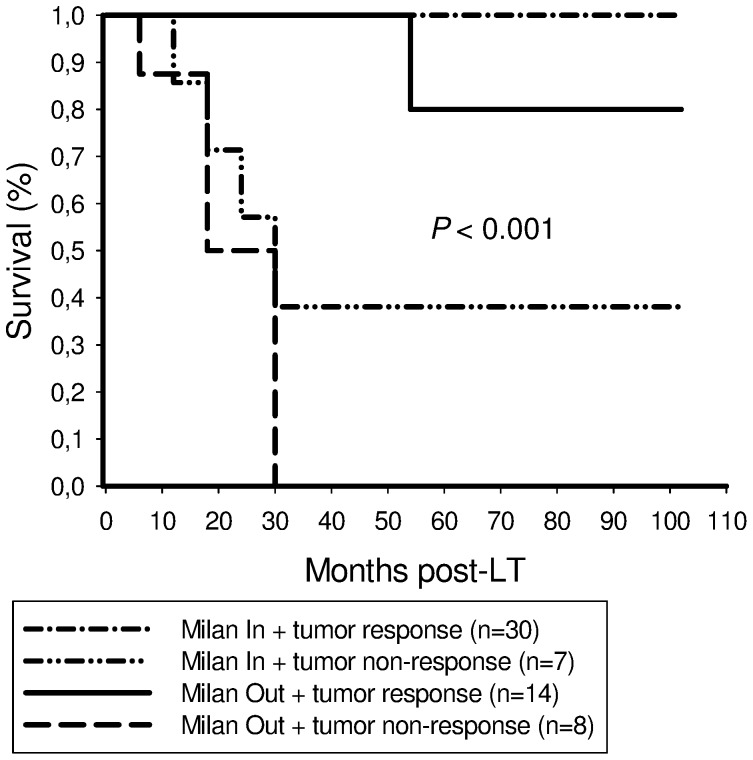
This graph illustrates outcome stratified by Milan criteria and post-IBT tumor response. We found excellent 5-year recurrence-free survival in Milan In and Milan Out patients with postinterventional tumor response (100%/80%), compared to inferior outcome in Milan In and Milan Out recipients without extended post-IBT tumor necrosis (38,1%/0%; *P*<0.001), respectively.

Five-year recurrence-free survival rates were 100% and 38.1% in Milan In patients, and 80% and 0% in Milan Out recipients with and without postinterventional tumor response, respectively (Fig. 6).

**Figure 7:**
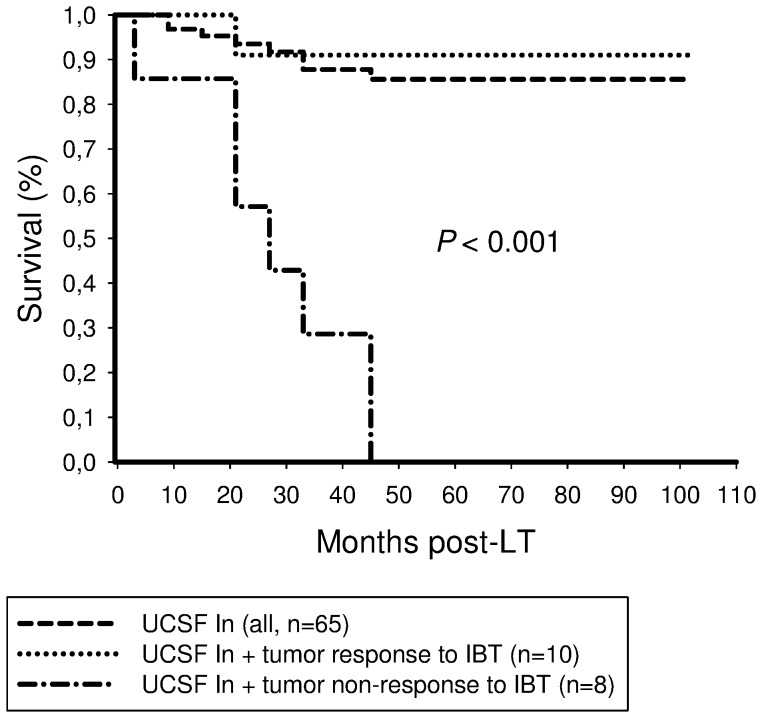
Five-year recurrence-free survival rate was 75% in UCSF Out patients exhibiting tumor response to IBT and 85.6% in UCSF In recipients of our trial, but 0% in UCSF Out patients without postinterventional tumor response, respectively (*P*<0.001).

Five-year tumor-free survival rates were 75% and 0% in UCSF Out patients with and without post-IBT tumor response, respectively (*P*<0.001; Fig. 7).

AFP level, number and total diameter of tumor nodules, Milan criteria, UCSF criteria, PET status, microvascular invasion, tumor differentiation, lymphatic and vascular invasion, and tumor response to IBT correlated significantly with outcome in the IBT subpopulation (Table 4). However, only tumor response to IBT was identified as independent predictor of freedom from tumor recurrence (Table 5).

**Table 4:** Univariate analysis of predictive parameters for posttransplant recurrence-free long-term-survival in the IBT group (n = 59).

*Variable*	*Log rank*
AFP level ≤100 IU/ml	<0.001
*Number of tumor nodules ≤3	0.008
*Total tumor diameter ≤10 cm	0.008
*Milan In status	0.02
*UCSF In status	<0.001
PET − status	<0.001
Microvascular Invasion	<0.001
G3-tumor	<0.001
Lymphatic invasion	<0.001
Tumor response to IBT	<0.001

* based on clinical staging.

PET: positron emission tomography.

G: grading.

AFP: alpha-fetoprotein.

UCSF: University of California San Francisco.

**Table 5:** Multivariate analysis of predictive parameters for posttransplant recurrence-free survival in the IBT group (n = 59).

*Variable*	*95% CI*	*HR*	*P-value*
Tumor response to IBT	6.66–426.19	53.3	<0.001

CI: confidence interval.

HR: hazard ratio.

### Pretransplant (clinical) variables predicting tumor response to IBT

AFP-level ≤100 IU/ml, number of tumor nodules ≤3 and negative pretransplant PET scans were predictive for tumor response to IBT in univariate analysis (Table 6). On multivariate assessment, only PET negativity of HCC was identified as independent clinical predictor of post-IBT tumor response (Table 7).

**Table 6:** Correlation of pretransplant available (clinical) parameters with tumor response to IBT (n = 59).

*Variable*	*Tumor response*	*No tumor response*	*P-value*
	(n = 44)	(n = 15)	
AFP level (n)			0.008
≤100 U/L	36	7	
>100 U/L	8	8	
*Number of tumor nodules (n)			0.05
≤3	39	10	
>3	5	5	
PET status (n)			<0.001
negative	36	4	
positive	8	11	

* based on clinical staging.

PET: positron emission tomography.

AFP: alpha-fetoprotein.

**Table 7:** Multivariate analysis of pretransplant available (clinical) parameters predicting tumor response to IBT (n = 59).

*Variable*	*95% CI*	*OR*	*P-value*
PET − status	3.1–49.0	12.4	<0.001

CI: confidence interval.

PET: positron emission tomography.

OR: odds ratio.

## Discussion

In the present study, our main goal was not to analyze the potential of IBT as downstaging modality prior LT but to assess its value for decreasing posttransplant risk of tumor recurrence, especially focusing on patients with advanced HCC on clinical staging. We were able to demonstrate that extended post-interventional tumor necrosis is an independent predictor of tumor-free long-term survival in liver transplant patients with HCC. Furthermore, our data point out that tumor response to IBT is a valuable indicator of favourable tumor biology and may predict excellent posttransplant outcome in patients with advanced HCC on pretransplant clinical staging.

Since the implementation of the Milan criteria in 1996, several transplant centers have advocated for LT as treatment in HCC beyond standard criteria [3]–[5]. New interventional options of pretransplant tumor treatment seem to support the idea of expanding transplant criteria. Yao et al. reported about recurrence-free survival benefits by neoadjuvant IBT after LT in the subgroup of pT2 and pT3 HCC [9]. Ravaioli et al. demonstrated that transplant criteria may be slightly extended without survival disadvantage after implementation of pretransplant IBT [15]. And recently, Chapman et al. showed that selected patients with stage III/IV HCC can be successfully downstaged and may, thereby, achieve excellent survival rates after LT [14]. In contrast, several other trials were not able to confirm these results [23]–[26]. Although liver-directed therapy is currently an accepted approach in the treatment of HCC, conclusive evidence of its efficacy in the transplant setting is still lacking [8]–[17], [24]–[26]. This may be related to differences in study design, listing criteria, pretransplant waiting times, and posttransplant surveillance concepts. Furthermore, the intended strategies of IBT were not consistent in recent trials. In some transplant centers IBT was implemented for tumor downstaging into accepted criteria before allowing LT [8], [14], [15], [25], [26]. Like others, we were not primarily using IBT for tumor downsizing but particularly for pretransplant tumor control in order to prevent patient drop-out and to decrease the risk of posttransplant tumor recurrence [11], [16], [25].

Apart from that, tumor-related drop-out criteria were not coherently defined. Macromorphological tumor progression beyond the listing criteria or beyond the proposed down-staged criteria excluded LT in many centers [15], [16], [25]–[27]. Such a waiting list policy reduces the risk of tumor recurrence, however for the price of an increasing rate of tumor-related drop-out and mortality [28], [29]. Contrary to this, tumor-associated patient de-listing was determined by biological rather than macromorphological tumor progression in our series [18], [20], [28], [30]. This may reduce the risk of drop-out but will increase the hazard of tumor recurrence post-LT. Decreasing vital tumor load prior to LT seems to be mandatory in this context. Nevertheless, the impact of IBT-induced tumor necrosis on recurrence-free outcome is very limited.

Postinterventional tumor response was identified as the only independent predictor of recurrence-free long-term survival related to IBT in our series (Table 4,5). Other established features of harmful tumor biology, such as microvascular invasion and poor tumor differentiation have lost their independent predictive power after including post-IBT tumor necrosis into our analysis. This is an interesting result of our study. Even though IBT capabilities to induce extensive tumor necrosis have already been demonstrated [13]–[17], [31]–[33], its impact on post-LT outcome is undefined. Five-year recurrence-free survival rate was not significantly different between patients receiving (78%) and those not receiving IBT (68%) in our series (Fig. 3), which is comparable to several other trials [25], [26]. This may be in part related to the retrospective study design and the relatively small sample size. However, from a scientific point of view, it appears to be inadequate to simply compare both populations. Liver transplant candidates who were directed to IBT represent a pre-selected subpopulation, since eligibility is determined by clinical condition, liver function and tumor morphology. For a better understanding of IBT capabilities in the transplant set-up, randomized controlled trials may be useful. From an ethical point of view, however, they are not to be expected, since effective antitumor treatment alternatives are still lacking. We have therefore stratified our data according to a histopathologic gold standard endpoint, which is missing in most other trials. Our results clearly indicate that extended tumor necrosis should be the major goal and posttransplant outcome variable of IBT in the transplant setting.

Furthermore, our data suggest that postinterventional tumor response may be a useful parameter of favourable tumor biology in patients with advanced HCC on pretransplant clinical imaging. Five-year recurrence-free survival rates were 80% and 75% in successfully treated Milan Out and UCSF Out patients, respectively (Fig. 5,6,7), which is completely comparable to Milan In patients. This extraordinary outcome data points out that a subset of patients with advanced HCC but with less aggressive tumor biology may achieve excellent posttransplant outcome, although exceeding standard criteria on pretransplant clinical staging. Based on results of our trial, this special subpopulation may not be excluded from LT, as it is, however, practice in many transplant centers. In contrast, if IBT failed to induce extended tumor necrosis, none of the patients with advanced HCC on radiographic imaging were still alive 5 years post-LT (Fig. 5,6,7). In the context of a dramatic shortage of appropriate donor organs, patients with such harmful tumor biology did retrospectively not qualify for LT [34]. Thus, it seems to be of great importance to identify pretransplant available clinical predictors of post-IBT efficacy in order to improve selection process beyond the Milan burden. Several imaging variables have been suggested to indicate postinterventional tumor necrosis on cross sectional radiography [35]–[37]. However, the experiences with correlating pre- and post-IBT imaging features of HCC nodules with histopathologic findings are still rather limited since only a minority of patients qualify for liver surgery. That is the reason why reliable radiographic features of post-IBT tumor necrosis have not been yet defined [35]–[37]. Rates of tumor understaging by preoperative imaging are still ranging between 20% and 40% [38], [39]. Furthermore, the value of radiographic TACE responsiveness to predict beneficial outcome in patients with advanced HCC is discussed controversially. Otto et al. suggested that TACE-induced progression-free follow-up could be an adequate clinical criterion for indicating LT in HCC beyond the Milan burden [13]. In contrast, TACE efficacy on radiographic imaging was not associated with improved outcome in Milan Out patients in a trial by Millonig et al. [40]. And just recently, Henry et al. demonstrated that best radiological response to TACE did not correlate with survival benefits in patients with unresectable HCC [41].

In our IBT patients, none of macromorphological variables defining the standard criteria but only negative PET status of HCC was identified as independent pretransplant available predictor of postinterventional tumor response (Table 6,7). Only 4 of 40 patients with ^18^F-FDG non-avid HCC (10%) emerged as tumor non-responders to IBT, compared to 11 of 19 patients with ^18^F-FDG avid tumors (57,9%; *P*<0.001). To the best of our knowledge, this interesting correlation has not been described before. Although sensitivity of ^18^F-FDG PET for diagnosing and detecting HCC is limited, it seems to have some importance for describing biological tumor behaviour in the transplant setting. Enhanced glucose metabolism on pretransplant PET was identified as useful clinical surrogate marker of poor tumor grading and microvascular invasion, which are both well-known parameters of tumor aggressiveness in HCC [18]–[20]. We have just recently demonstrated that patients with PET negative advanced HCC may achieve excellent outcome post-LT [30]. According our current data, this may be triggered by extensive postinterventional tumor necrosis, as none of 10 patients with PET negative (0%), but 7 of 12 patients with PET positive HCC exceeding the Milan criteria (58,3%) have developed tumor recurrence post-LT, respectively. Five liver transplant patients with PET positive advanced HCC remained free from tumor recurrence and, noteworthy, 4 of them revealed extended tumor necrosis at explant pathology. If postinterventional tumor response might have been indicated in these patients by a switch from PET + status pre-IBT to PET − status post-IBT can not be answered by our data, since we did not routinely perform follow-up PET scans. This is a drawback of our trial.

There are some further limitations in our study. First, tumor response data were assessed retrospectively and the sample size was rather small. Furthermore, we have included patients after different interventional procedures, although TACE was the predominantly used locoregional intervention in our series. In contrast, our data is powered by a concise prospective clinical follow-up in a relatively high number of patients with advanced HCC on radiographic imaging. Moreover, we have used clinical but not histopathologic features of the Milan/UCSF criteria for uni- and multivariate assessments.

In conclusion, we were able to demonstrate that extended postinterventional tumor necrosis is an indicator of favourable tumor biology in the transplant setting. It promotes recurrence-free long-term survival in liver transplant patients with HCC beyond standard criteria on clinical imaging. Post-LT tumor surveillance should be intensified in patients without tumor response to IBT, since risk of tumor recurrence is significantly increased. Pretransplant PET evaluation seems to be useful in identifying those patients with HCC beyond standard criteria that may benefit from IBT and, thereby, achieve an excellent long-term survival. If postinterventional tumor response in patients with PET positive tumors may be indicated by a decrease of ^18^F-FDG-uptake patterns on PET has to be analyzed in a prospective approach.
